# Management of Delayed Eruption of Permanent Maxillary Incisor associated with the Presence of Supernumerary Teeth: A Case Report

**DOI:** 10.5005/jp-journals-10005-1121

**Published:** 2011-04-15

**Authors:** Naveen Manuja, Rajni Nagpal, Mousumi Singh, Seema Chaudhary

**Affiliations:** 1 Reader, Department of Pediatric Dentistry, Kothiwal Dental College, Moradabad, Uttar Pradesh, India; 2 Reader, Department of Conservative Dentistry, Kothiwal Dental College, Moradabad, Uttar Pradesh, India; 3 Professor and Head, Department of Pediatric Dentistry, Kothiwal Dental College, Moradabad, Uttar Pradesh, India; 4 Professor, Department of Pediatric Dentistry, Kothiwal Dental College, Moradabad, Uttar Pradesh, India

**Keywords:** Delayed eruption, Maxillary left central incisor, Supernumerary teeth.

## Abstract

A supernumerary tooth is one that is additional to the normal series and can be found in almost any region of the dental arch. Clinically, supernumerary teeth are able to cause different local disorders. It is important for the dentist to be aware of the clinical complications of supernumerary teeth, the most common being the delayed eruption of permanent teeth. Early diagnosis and management of supernumerary teeth is important to prevent the need for more complex surgical and orthodontic treatment. This case report highlights the problem of delayed eruption of permanent maxillary left central incisor in a 9-year-old boy due to two supernumerary teeth, one tuberculate type and other impacted inverted mesiodens.

## INTRODUCTION

Several causes of delayed eruption of permanent maxillary incisors have been reported in the literature such as supernumerary teeth, tooth agenesis, tooth malformation or dilacerations, cysts or other pathological obstructions in the eruptive path, presence of a dense mucoperiosteum or submucosa that acts as a physical barrier to eruption, retained primary incisor that has become ankylosed, lack of space or in association with certain syndromes.^1^ However, the presence of supernumerary teeth in the premaxillary region has been cited as the most common cause for delayed eruption of permanent maxillary incisors.^2-4^

Supernumerary teeth are defined as excess in the number of teeth when compared with the normal dental formula.^5^ They are more prevalent in the permanent dentition with reports of between 1 and 3% of the general population affected, while in the primary dentition they are found in approximately 0.8% of the population.^6-8^ A sex-linked mode of inheritance has also been suggested, as supernumerary teeth are twice as common in males as females in the permanent dentition.^9,10^ Approximately, 90% of supernumerary teeth are found in the maxilla.^5^

The etiology of supernumerary teeth remains unknown; however, it is appropriate to consider hyperdontia as a multifactorial inheritance disorder.^11^ While the occurrence of supernumerary teeth cannot be predicted, the influence of genetic factors is strongly suggested. Several theories have been put forward concerning the cause of this dental anomaly, such as the phylogenetic theory,^12^ the dichotomy theory,^13^ a hyperactive dental lamina^5,14^ and a combination of genetic and environmental factors.^14^ The most widely accepted of these is the hyperactivity theory, which implies that supernumerary teeth are the result of excessive but organized growth of the dental lamina. Remnants of the dental lamina or palatal extensions of the active dental lamina are induced to develop into an additional tooth bud, which results in a supernumerary tooth. The presence of supernumerary teeth may be part of developmental disorders such as cleft lip and palate, cleidocranial dysostosis, Gardner’s syndrome, Fabry Anderson’s syndrome, Ellis Van Creveld syndrome (chondroectodermal dysplasia), Ehlers-Danlos syndrome, in-continentia pigmenti and trichorhinophalangeal syndrome.^15^

Supernumerary teeth can be classified according to their location in the dental arch as mesiodens, paramolar and distomolar or according to their morphology as conical, tuberculate, supplemental and odontome.^10^ The term mesiodens (coined by Bolk) denotes supernumerary teeth located in the midline of the maxilla between the central incisors.^16-18^ The overall prevalence of mesiodens is between 0.15 and 1.9%.^5,19,20^ A paramolar most commonly occurs in the interproximal space buccal to the maxillary second and thirdmolars; and a distomolar is a fourth permanent molar which is usually placed either directly distal or distolingual to the third molar. Conical type of supernumerary tooth is peg-shaped and develops with root formation ahead of or at an equivalent stage to that of permanent incisors and usually presents as a mesiodens. It may occasionally be found high and inverted into the palate or in a horizontal position. It can result in rotation or displacement of the permanent incisor, but rarely delays eruption.^21^ The tuberculate type of supernumerary possesses more than one cusp or tubercle. It is frequently described as barrel-shaped and may be in-vaginated. Root formation is delayed compared with that of the permanent incisors resulting in a rudimentary root. They rarely erupt and are frequently associated with delayed eruption of the incisors.^21^ The supplemental supernumerary refers to a duplication of teeth in the normal series and is found at the end of a tooth series.

The clinical complications of supernumerary teeth include delayed eruption of permanent incisors, midline diastema, axial rotation or inclination of erupted permanent incisors, resorption of adjacent teeth, root anomaly and cyst formation.^22^ Here, we present a case report of two supernumerary teeth in the anterior maxillary region―one erupted tuberculate type and other impacted inverted mesiodens causing delayed eruption of permanent left maxillary central incisor.

## CASE REPORT

**Fig. 1:**
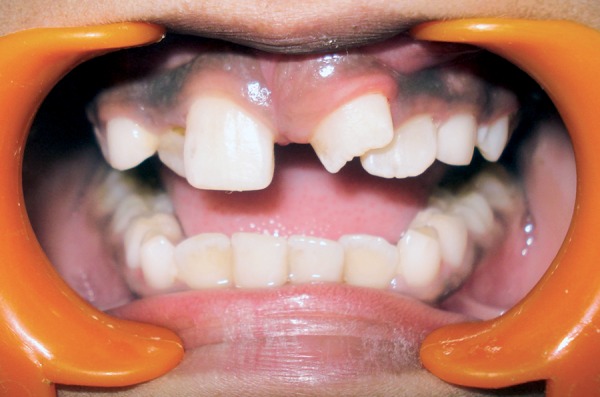
Intraoral view showing partially erupted and rotated tooth in the position of left maxillary permanent central incisor

**Fig. 2:**
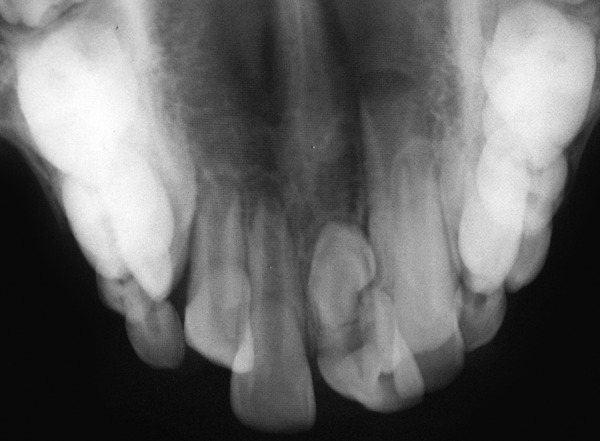
Occlusal radiograph illustrating two supernumerary teeth― one erupted and other impacted inverted mesiodens and impacted permanent left maxillary central incisor with immature root

**Fig. 3:**
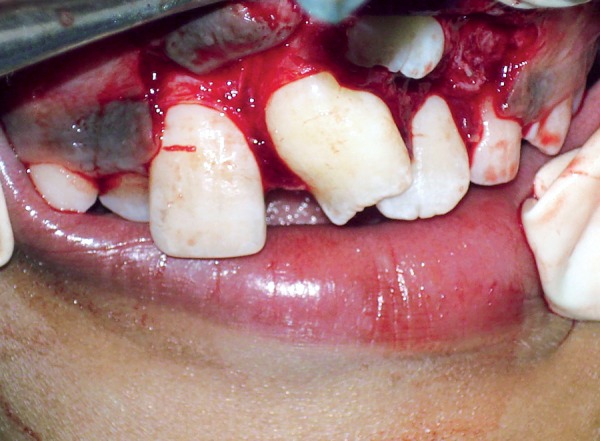
Impacted permanent central incisor visible after raising buccal flap

**Fig. 4:**
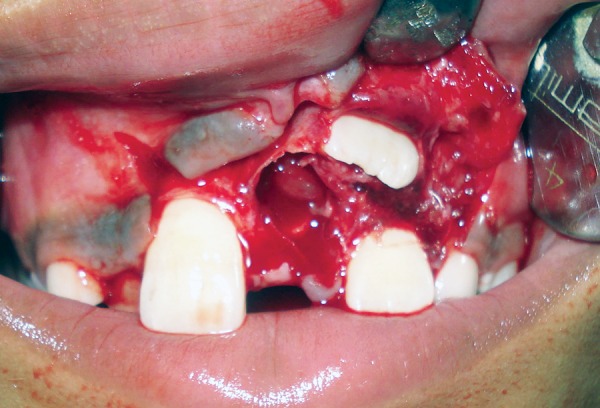
Surgical site after extraction of erupted supernumerary tooth

**Fig. 5:**
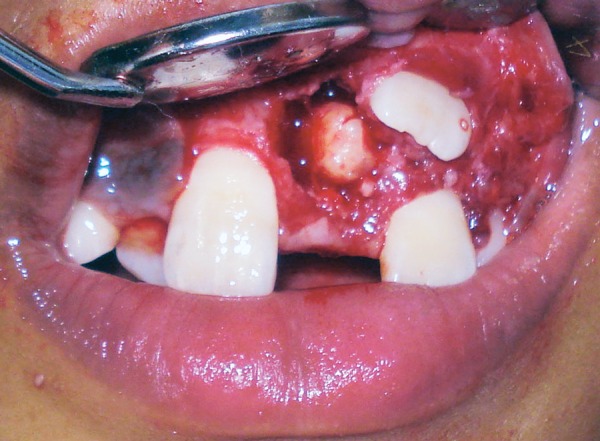
Surgical exposure of impacted inverted mesiodens

**Fig. 6:**
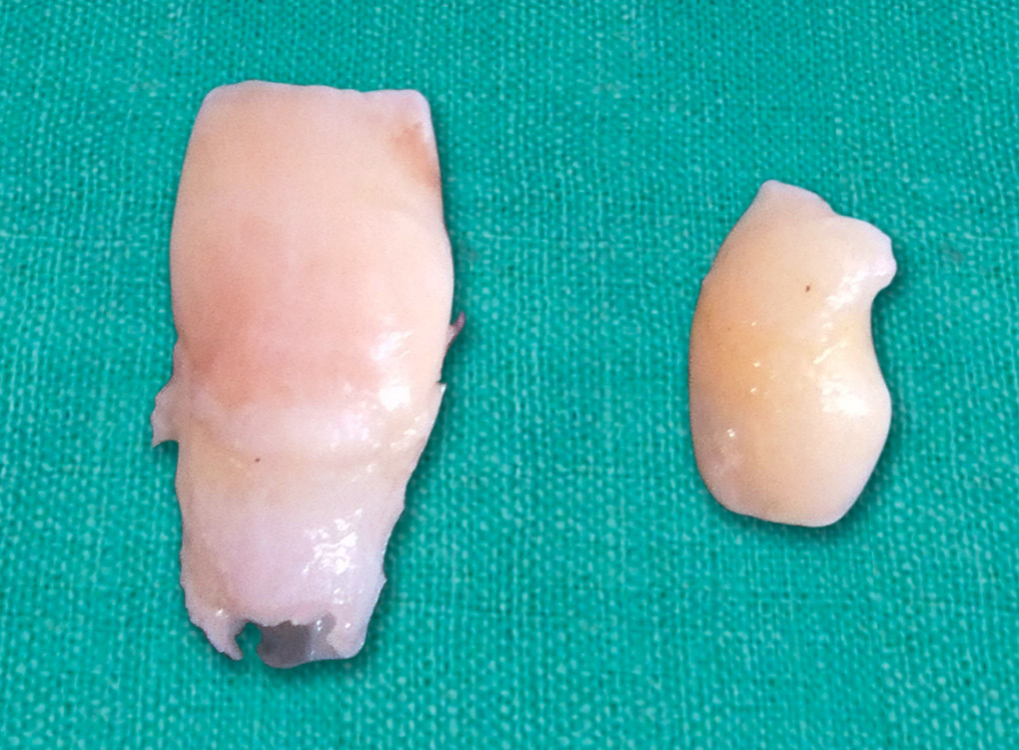
Supernumerary teeth after removal

**Fig. 7:**
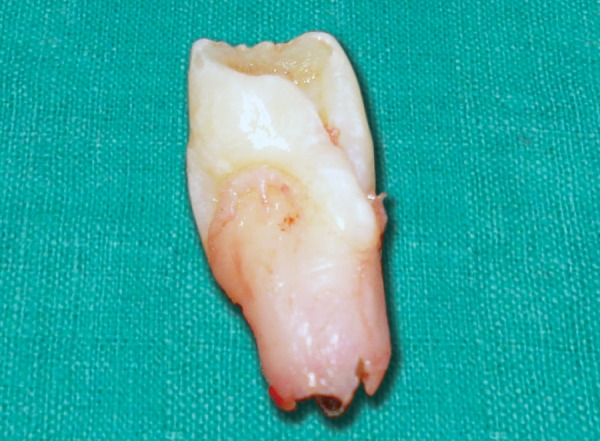
Tuberculate supernumerary tooth showing invagination palatally

**Fig. 8:**
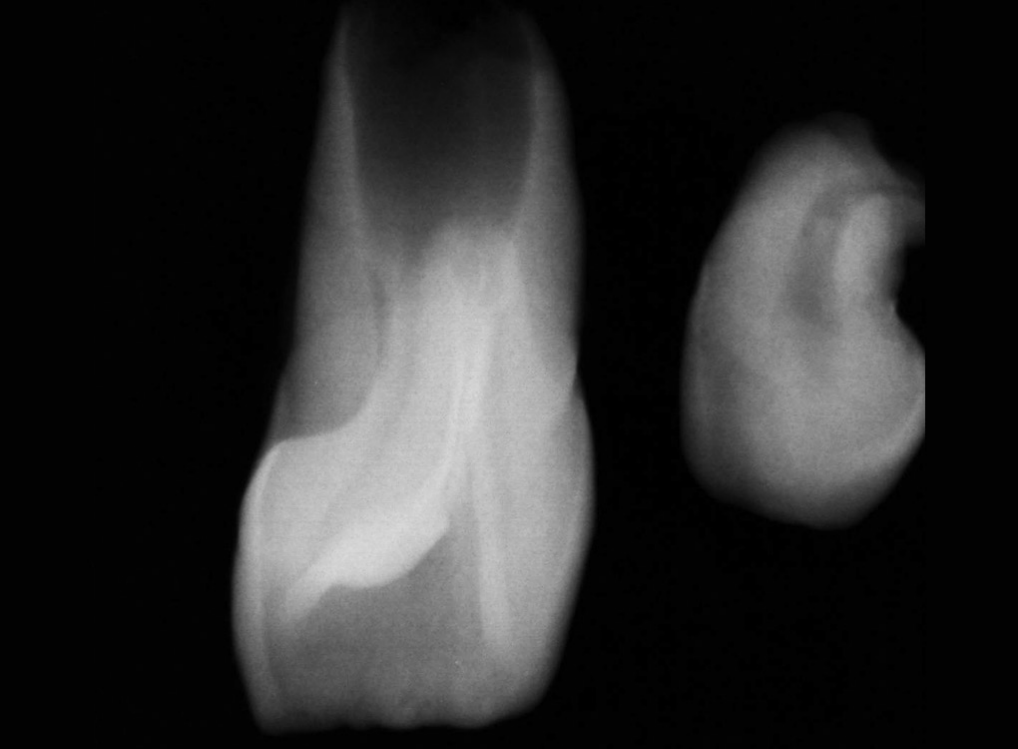
Radiograph of supernumerary teeth depicting invagination in the tuberculate type

**Fig. 9:**
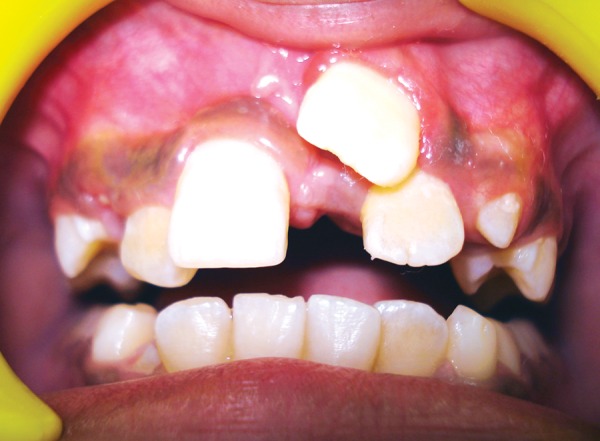
Six-month postoperative clinical photograph reveals erupting left permanent maxillary central incisor

**Fig. 10:**
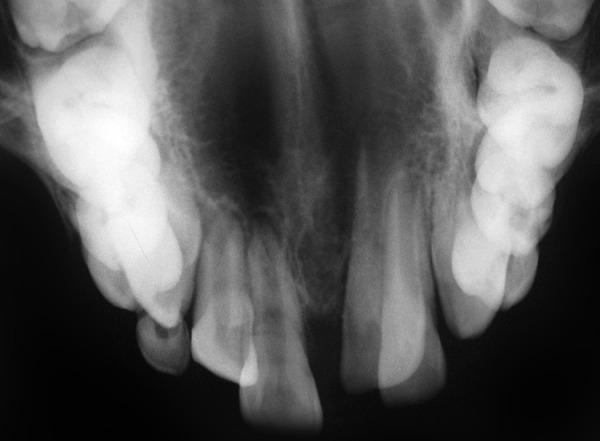
Six-month postoperative occlusal radiograph showing occlusal movement of left permanent maxillary central incisor

A 9-year-old boy reported to the Pediatric Dental Clinic for routine dental check-up. His medical history was unremarkable and there was no family history of supernumerary or congenitally missing teeth. Intraoral examination revealed Class I mixed dentition and partially erupted and rotated tooth in the position of left maxillary permanent central incisor (Fig. 1). Radiographic examination depicted un-erupted left maxillary permanent central incisor with immature root and two supernumerary teeth―one erupted and other impacted inverted mesiodens (Fig. 2). The need for removal of both supernumerary teeth was explained to the patient and parents to facilitate the eruption of permanent left central incisor. Surgical procedure was carried out under local anesthesia. The buccal flap was raised and the impacted permanent left central incisor was visible (Fig. 3). Erupted supernumerary tooth was extracted (Fig. 4). After this, some portion of bone was removed using slow speed bur with copious saline irrigation to expose the impacted inverted mesiodens (Fig. 5). This inverted mesiodens were removed. The margins of the bone were smoothened. The flap was sutured and hemostasis was achieved. Visual examination revealed that both supernumerary teeth had incompletely formed root (Fig. 6). The extracted supernumerary tooth was invaginated palatally (Fig. 7). Radiograph of this tooth revealed the presence of invagination that appeared to be lined by enamel (Fig. 8). This tooth appeared to be of tuberculate type supernumerary. Six month follow-up clinical and radiographic examination revealed erupting permanent left maxillary central incisor (Figs 9 and 10).

## DISCUSSION

Supernumerary teeth frequently interfere with the eruption and alignment of the maxillary incisors.^2,23,24^ They can delay or prevent eruption of central incisors in 26 to 52% of cases; cause ectopic eruption, displacement or rotation of a central incisor in 28 to 63% of cases; and labially displace incisors in 82% of cases.^5,23^ Less common complications involving the permanent incisors include dilaceration of the developing roots, root resorption and loss of tooth vitality. Complications involving the supernumerary tooth itself include eruption of the supernumerary tooth into the nasal cavity and development of a dentigerous cyst which has been reported in 4 to 9% of cases.^25-30^

Most supernumerary teeth remain impacted, but in approximately 25% of cases eruption occurs.^11^ Diagnosis is best achieved by thorough clinical and radiographic examination. Buccolingual position of unerupted supernumeraries can be determined using the parallax radiographic principle.^1,31^ Maxillary occlusal radiography is highly recommended for all children with dental disturbances in the premaxilla.^18^ Once a supernumerary tooth has been diagnosed, the clinician must decide on treatment to minimize further sequel. To avoid the complications of supernumerary teeth, extraction of these teeth is a general rule. Timing of surgical removal of supernumerary teeth has also been contentious. Two alternatives exist:^32^ The first option involves removal of the supernumerary as soon as it has been diagnosed. This could create dental phobia problems for a young child and has been said to cause devitalization or deformation of adjacent teeth. Second, the supernumerary could be left until root development of the adjacent teeth is complete. The potential disadvantages associated with this deferred surgical plan include; loss of eruptive force of adjacent teeth, loss of space and crowding of the affected arch, and possible midline shifts.

According to Solares,^33^ extraction during the early mixed dentition stage allows normal eruptive forces to promote spontaneous eruption of the permanent central incisors following extraction. Hogstrom and Andersson^32^ suggested that early interventions are preferable to take advantage of the spontaneous eruption potential of the permanent incisors and to prevent anterior space loss and midline deviation. Extraction of supernumerary tooth at a time appropriate for promoting self-eruption in the early mixed dentition may result in better alignment of the teeth and may minimize the need for orthodontic treatment.

Delayed treatment involves extraction of the supernumerary tooth when the unerupted central incisor’s apex is almost mature.^34^ The later the extraction of the supernumerary tooth, the greater the chance that the permanent tooth either will not spontaneously erupt or will be malaligned when it does erupt. Unfortunately, by this time the forces that cause normal eruption of the incisors are diminished, and surgical exposure and subsequent orthodontic treatment are more frequently required.^29^ Also, space loss and a midline shift of the central incisors may have already occurred by this age, since the lateral incisors will have erupted and may have drifted mesially into the central space.^11^ Thus, a significant delay in treatment can create the need for more complex surgical and orthodontic management.

Management of the delayed eruption of a tooth due to a supernumerary can be approached by any one of the following three methods:^3^ First, by conservative management, by removal of the supernumerary only. Second, by removal of the supernumerary tooth together with the bone overlying the unerupted tooth with or without placement of a bonded attachment for orthodontic traction and replacement of the flap (closed exposure). And third, by removal of the supernumerary and exposure of the unerupted tooth, with or without placement of a bonded attachment for orthodontic traction (open exposure).

Close monitoring of the dentition is required after the extraction of a supernumerary tooth. In 75% of cases, the incisor erupts spontaneously once the supernumerary tooth has been removed.^2,26^ Mitchell and Bennett^3^ studied spontaneous eruption following supernumerary removal only. They found that 78% spontaneously erupted with a median time for eruption of 16 months. Di Biase^26^ and Leyland et al^35^ reported that most teeth experiencing delayed eruption will spontaneously erupt within 18 months of supernumerary removal alone. Mason et al^36^ observed that more immature teeth erupted spontaneously after removal of the supernumerary compared with mature teeth.

In the case presented, the possibility of spontaneous eruption after removal of supernumerary teeth was explained to the parents and their consent was taken. After initial follow-up of 3 months, a monthly follow-up was planned. Six months postoperative clinical examination reveals erupting permanent left maxillary central incisor (Fig. 9) and in the radiographic examination occlusal movement of this tooth is evident with still immature roots (Fig. 10). The need for the fixed orthodontic treatment after complete eruption of the permanent teeth was explained to the patient for the correction of malocclusion.
